# Mental Distress and Human Rights Violations During COVID-19: A Rapid Review of the Evidence Informing Rights, Mental Health Needs, and Public Policy Around Vulnerable Populations

**DOI:** 10.3389/fpsyt.2020.603875

**Published:** 2021-01-08

**Authors:** Muhammad Rahman, Rabab Ahmed, Modhurima Moitra, Laura Damschroder, Ross Brownson, Bruce Chorpita, Priscilla Idele, Fatima Gohar, Keng Yen Huang, Shekhar Saxena, Joanna Lai, Stefan Swartling Peterson, Gary Harper, Mary McKay, Beatrice Amugune, Tammary Esho, Keshet Ronen, Caleb Othieno, Manasi Kumar

**Affiliations:** ^1^University of Washington, Seattle, WA, United States; ^2^Washington University, St. Louis, MO, United States; ^3^Department of Health Metrics Sciences, University of Washington, Seattle, WA, United States; ^4^VA Ann Arbor Healthcare System, Ann Arbor, MI, United States; ^5^Department of Psychology, University of California Los Angeles Life Sciences, Los Angeles, CA, United States; ^6^United Nations International Children's Emergency Fund (UNICEF), New York, NY, United States; ^7^New York University, New York, NY, United States; ^8^Harvard T.H Chan School of Public Health, Harvard University, Cambridge, MA, United States; ^9^Uppsala University, Uppsala, Sweden; ^10^University of Michigan, Ann Arbor, MI, United States; ^11^University of Nairobi, Nairobi, Kenya; ^12^Amref Health Africa, Nairobi, Kenya; ^13^University of Botswana, Gaborone, Botswana

**Keywords:** mental and behavioral health, human rights, lockdown, health care worker [non-MESH], stigma and discrimination, vulnerable populations, LMICs (low and middle income countries)

## Abstract

**Background:** COVID-19 prevention and mitigation efforts were abrupt and challenging for most countries with the protracted lockdown straining socioeconomic activities. Marginalized groups and individuals are particularly vulnerable to adverse effects of the pandemic such as human rights abuses and violations which can lead to psychological distress. In this review, we focus on mental distress and disturbances that have emanated due to human rights restrictions and violations amidst the pandemic. We underscore how mental health is both directly impacted by the force of pandemic and by prevention and mitigation structures put in place to combat the disease.

**Methods:** We conducted a review of relevant studies examining human rights violations in COVID-19 response, with a focus on vulnerable populations, and its association with mental health and psychological well-being. We searched PubMed and Embase databases for studies between December 2019 to July 2020. Three reviewers evaluated the eligibility criteria and extracted data.

**Results:** Twenty-four studies were included in the systematic inquiry reporting on distress due to human rights violations. Unanimously, the studies found vulnerable populations to be at a high risk for mental distress. Limited mobility rights disproportionately harmed psychiatric patients, low-income individuals, and minorities who were at higher risk for self-harm and worsening mental health. Healthcare workers suffered negative mental health consequences due to stigma and lack of personal protective equipment and stigma. Other vulnerable groups such as the elderly, children, and refugees also experienced negative consequences.

**Conclusions:** This review emphasizes the need to uphold human rights and address long term mental health needs of populations that have suffered disproportionately during the pandemic. Countries can embed a proactive psychosocial response to medical management as well as in existing prevention strategies. International human rights guidelines are useful in this direction but an emphasis should be placed on strengthening rights informed psychosocial response with specific strategies to enhance mental health in the long-term. We underscore that various fundamental human rights are interdependent and therefore undermining one leads to a poor impact on the others. We strongly recommend global efforts toward focusing both on minimizing fatalities, protecting human rights, and promoting long term mental well-being.

## Introduction

On March 11, 2020, the World Health Organization (WHO) declared a public health emergency of international concern in response the global pandemic of the novel Coronavirus disease (SARS-COV-2). To reduce the spread of the virus, countries have implemented urgent emergency health measures. These measures include stay at home orders and the closure of schools which have led people to reorganize their lives and necessitated changes in livelihood and health services (1, 2).

In responding to public health emergencies, governmental authorities have to navigate the delicate balance between protecting the public's health and safeguarding their inherent human rights including education, freedom of movement, and access to health care. Measures to prevent the spread of infectious diseases are not zero-sum tradeoffs and can decrease fatalities but also increase suffering if human rights are not respected. As such, while being protected from clear public health threats, many people, especially vulnerable populations, may be deprived of their inherent human rights (3). We are in favor of using science to achieve globally shared objectives, but it is important to consider all sources of evidence in addition to the infectious diseases realm in the contexts of known tradeoffs—between lockdown and freedom to assert social and economic freedom. We recommend nations focus both on minimizing fatalities and protecting human rights. The United Nations (UN) defines human rights as: “*…fundamental to all human beings, regardless of race, sex, nationality, ethnicity, language, religion, or any other status. These rights include the right to life and liberty, freedom from slavery and torture, freedom of opinion and expression, the right to work and education, and many more such as a safe & clean environment have become important to uphold. Everyone is entitled to these rights, without discrimination or threat of any kind”* (4).

Within bioethics, the needs of different populations marked as vulnerable have even more urgent and impactful implications because applying the human rights lens focuses on highlighting and protecting the rights withing vulnerable populations. Vulnerable populations are defined as “*groups and communities at a higher risk for poor health as a result of the barriers they experience to social, economic, political and environmental resources, as well as limitations due to illness or disability”* (5). The principle of vulnerability is pertinent in the context of global disasters. It emphasizes ethical discourse on ameliorating the conditions that produce vulnerability, rather than on emergency actions focused on saving lives (6). Vulnerability is a global phenomenon but in the context of the pandemic it is exacerbated in many societies where inequality and access to basic freedoms is restricted. In lower- and middle-income countries (LMICs) such freedoms can easily be impacted due to existing system challenges and adverse social determinants of health. In High Income Countries (HICs), similar challenges surface during pandemic, and vulnerable groups have also been systematically marginalized.

Mental health is integral, closely related to, and dependent upon the realization of human rights (7). In the context of the coronavirus disease 2019 (COVID-19) pandemic, reports highlight gross undermining of mental health and violations of individual civil liberties and fundamental rights such as mobility rights, access to accurate information, access to proper protection for health workers, right to education, and discrimination against marginalized populations (8). Human rights protection and mental health needs are not always adequately integrated into emergency response policy and management. The evidence highlights that vulnerable populations, including those living in abusive families, individual with disabilities, children, elderly, domestic caregivers, health care workers (HCWs), and ethnic and marginalized communities, are especially at risk for mental health distress (9–11) with the psychological needs of these populations not likely to be fulfilled without meaningful legislation and intervention (9, 12). There have been long standing concerns within the field of mental health that the human rights of psychiatric populations and individuals and communities under psychosocial distress tend to be ignored. From mental health policy perspectives, how human right policies and practices is integrated in emergency response policy and management has not been focused. There are several aspects of vulnerability, disability, coerciveness of treatment and system level oppression that have been key concerns. In this review we focus on rights associated violations and grievances that would potentiate further mental distress and long-term harm to critical subsections of at-risk populations that will be negatively impacted.

Given the current evidence, we believe that it is important to broaden our understanding of what rights violations entail in the context of this global pandemic and learn critical lessons in mitigation and prevention of these abusive and unethical situations and mental distress emanating from these. As the first step to inform global human rights policy and guidelines, and practices for better managing a global pandemic, it is important to understand and summarize current knowledge and practices. The focus of this review therefore is to collate evidence on human rights abuses and violations and resultant mental and behavioral health outcomes of different populations, especially those known to be at high risk or have greater vulnerabilities warranting additional mitigation, prevention, and treatment approaches during COVID-19 (13). The two specific objectives are:

1) Scoping of prominent rights-based issues with a focus on basic human rights violations during the COVID-19 pandemic and its association with mental and behavioral health of populations. In this regard, people from different ages, regardless of their gender, ethnicity, status (i.e., orphans or refugees), profession, or religion, will be included.
2) Appraisal of binding agreements and global or national policies that enforce human rights during emergencies and developed for this pandemic that are designed to strengthen human rights and well-being of vulnerable populations.

## Methods

A rapid literature review was conducted using published data sources on human rights violations and resulting psychological impact on vulnerable populations during COVID-19. For this review, we used the following working definition of rapid review “*…a type of knowledge synthesis in which components of the systematic review process are simplified or omitted to produce information in a short period of time”* (14, 15).

Literature that focused on human rights and related violations, health stigma and discrimination in the context of the pandemic was prioritized. For mental health, literature related to both generic factors such as quality of life, well-being and condition-specific aspects such as symptoms due to human rights abuses were included. The search strategy involved locating relevant concepts in PubMed and Embase databases. Relevant data was extracted and summarized into five main themes: (1) Mobility rights, quarantine, and lockdown, (2) Shortage of supplies and equipment for HCWs, (3) Child rights, (4) Elderly's rights, and (5) Disproportionate impacts on minority rights and psychiatric patients.

A search was conducted in July 2020 for studies published during December 2019 to July 2020 with assistance from a health science librarian. After eliminating duplicates, we screened titles and abstracts (MR and MK) and did a secondary screening of full-text articles using the inclusion/exclusion criteria and extracted data (MR, RA, and MK). A cursory search of Google Scholar was also conducted to identify any possible missed studies that meet the inclusion criteria. All authors read and commented on human rights and mental health related literature and their associations. We have reported findings following the PRISMA guidelines (16).

**Figure 1:**
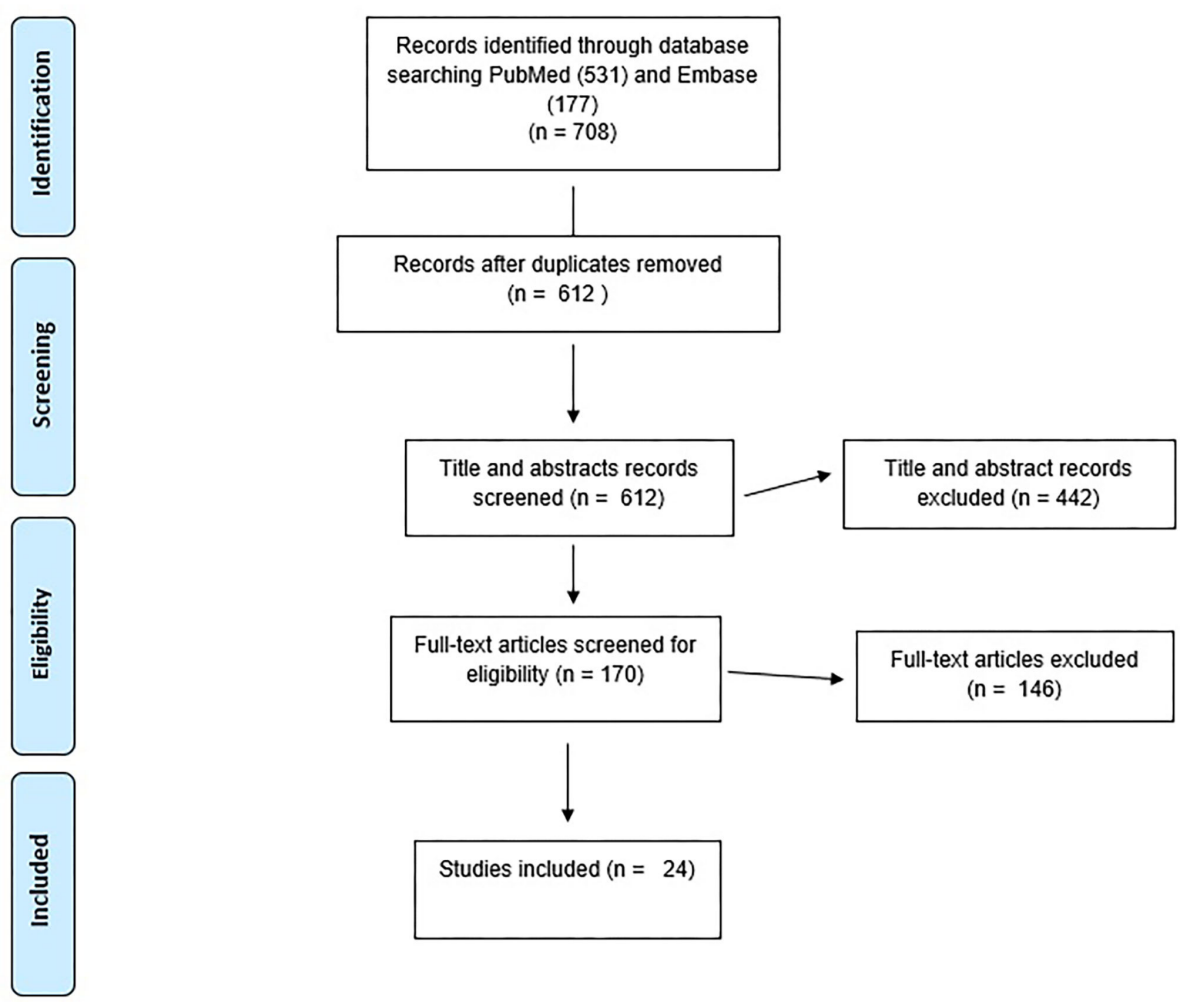
PRISMA flow diagram.

Studies were included if they described an empirically based (data-driven either qualitatively or quantitatively) assessment of human rights violations, social stigma and discriminatory behaviors against people of certain ethnic backgrounds, HCWs, and anyone perceived to have been in contact with the virus. It also included studies that reported mental, social, and behavioral health outcomes of the violations according to DSM/ICD classifications diagnostic categories or as ascertained using psychometric instruments. Furthermore, protocols and reports including guidelines by international agencies vetted by scientific and peer reviewers which address one of the primary outcomes were included. Studies were excluded if they tested one of the primary outcomes of interest without mentioning the association between them or the association between the constructs of interest or if those were not assessed in a data-driven approach. Systematic reviews, meta-analysis or scoping reviews were excluded if the analysis did not include one of the primary outcomes. Studies that are not peer-reviewed articles or not published in the English language were also excluded. Figure 1 shows the flowchart diagram of the selection of articles.

## Results

A total of 24 studies were included in this review (see Figure 1). There was a wide variation in the included studies between methodology, the study design, and location. These studies represented findings from 14 countries (six from HICs and eight LMICs). Based on their methodological approach, 14 used a cross-sectional study design, three were observational studies, two were case studies and the rest had differing study designs such as two being case controls.

Of the empirically based studies, five studies examined impacts of limited mobility rights, due to quarantine and lockdown, on mental health; four studies examined impacts of lack of PPE or stigma on HCWs; and the rest examined impacts of COVID-19 such as violation of right to education, health care, and access, on special populations, including children, elderly, minorities, and psychiatric patients. The sections below highlighted key results in each area. Table 1 describes the characteristics for articles reviewed under each area (e.g., country, theme of violation, study population, design, and findings).

**Table 1:** Included studies.

**Country and authors**	**World bank income level**	**Theme of violation**	**Type of population**	**Key findings**	**Study design**
Colombia (29)	Upper-middle income	Mobility restrictions and lack of adequate resources for mental health support and suicide intervention	General (ages 18–76)	•7.6% of participants reported a high suicide risk. 1 out of 13 Colombians in a non-probability sample reports a high suicide risk during COVID-19 •association between perceived stress related to COVID-19, risk of depressive episode, insomnia, and suicidal behavior in the context of a restriction on the mobility	Cross-sectional
Pakistan (30)	Lower-middle income	Economic hardships including unemployment due to mobility restrictions without access to financial support caused by lockdown	Suicide cases (ages 24–68)	•Most of the suicide cases occurred due to the lockdown-related economic recession •24% of population lives below poverty line and 20.5% is undernourished, both issues exacerbated by lockdown	Retrospective cohort suicide research
India (31)	Lower-middle income	Misinformation and fear or stigma related to possible COVID-19 infection were primary causes of suicide	Suicide attempts and cases (ages 18 and above)	•Rise in the number of suicide cases, which coincides with an increase in confirmed cases of COVID-19 and mitigation efforts (e.g., countrywide lockdown) •Social, financial insecurity, loss of employment or business, increase in family disputes, and pre-existing mental illness or medical illness might be predisposing and precipitating factors for an increase in suicide cases	Retrospective suicide research
United Kingdom (32)	High income	Significant psychological symptoms caused by lockdown but majority could not or did not receive formal or informal support indicating need for intervention and support especially for vulnerable populations and victims of gender based violence	General	•9% surveyed reported experiencing psychological or physical abuse, 18% reported experiencing thoughts of suicide or self-harm in the first month of lockdown and 5% reported harming themselves at least once •The reported frequency of abuse, self-harm and thoughts of suicide/self-harm was higher among women, black, Asian and minority ethnic groups and people experiencing socioeconomic disadvantage, unemployment, disability, chronic physical illnesses, mental disorders and COVID-19 diagnosis •Psychiatric medications most common type of support, but fewer than half of those affected were accessing formal or informal support	Analysis of COVID-19 Social Study: a non-probability sample weighted to population proportions
United States (33)	High income	Worse health outcomes in part due to knowledge gaps in effective prevention methods and inability to adhere to prevention methods due to crowding and work conditions	General, minority	•Black and Hispanic populations bear a disproportionate burden of medical conditions across their lifespans, including obesity, diabetes, and heart disease •COVID-19 disparities exacerbated by structural racism, which impacts housing, economic opportunities, education, transportation, food availability, and health care access	Cross-sectional survey
India (34)	Lower middle income	Misinformation, rumormongering, and negative perceptions influenced by media worsen marginalization, prejudice and stigma harming both targets and those with prejudiced viewpoints	General (ages 18–83)	•Fear of COVID-19, age, collectivism, and generalized xenophobia are closely linked with well-being •Significant negative relationship between fear of COVID-19 and well-being indicating holding xenophobic attitudes may be harmful not just for target of prejudice but also for those holding such attitudes •Critical to prevent marginalization and lessen COVID-19 stigma via proper information being distributed and combating misinformation	Cross-sectional survey
India (35)	Lower-middle income	Worry of length of lockdown and impacts on livelihood including financial troubles due to inadequate and unclear support	Migrant workers	•Difficulties in following precautions such as frequent hand washing, maintaining social distancing within shelter-mates and wearing masks •Uncertainty about the duration of lockdown, eagerness to travel and meet family, fear of being abandoned/deserted by employers, insecurity over income and job, substance use–related concerns •Pregnant women and children particularly feared inattention to their health issues	Qualitative—in person visits by mental health professionals
Jordan (36)	Upper-middle income	Strict lockdown, spread of misinformation, fear of contracting and spreading COVID and lack of PPE contributed to anxiety symptoms	General population, HCWs (ages 18 and above)	•Anxiety prevalent by health care professionals was 11.3% compared to general population 8.8% •Females among health care professionals, pulmonologists and ENT specialists at front lines, at higher risk of developing depression •Contributing factors are physician burnout, isolation from family, and feeling helpless due to the nature of this disease	cross-sectional survey
Lebanon (37)	Upper-middle income	Heavy psychological stress due to isolation from family support due to possible COVID infection, worry over stigma, and frustration over unclear information and health policy	HCWs: 13 quarantined health care professionals, 9 (69.2%) nurses 4 (30.8%) physicians	•HCWs being psychologically challenged through quarantine and need clear health communication from nursing managers, leaders, and policymakers •Proper health communication should be provided to the public so that they can know the reality of the situation and bypass any misconceptions and stigmatization •Moral and financial support should be offered to the quarantined personnel from governmental and non-governmental health policymakers	Qualitative design
Singapore (38)	High income	Perceived stigma against HCWs due to increased risk of infection lead to avoidance of public and worsening mental health outcomes indicating need for combatting stigma and stress	HCWs: residents with 61.7% junior and 38.3% senior	•No differences found between junior and senior residents in psychological and coping responses to pandemic •Those deployed to high-risk areas had lower perceived stress as those not deployed to high-risk areas had anticipatory anxiety contributing to higher stress levels •Higher perceived stigma level associated with higher levels of perceived stress and post-traumatic stress symptoms	Cross-sectional survey
Egypt (39)	Lower-middle income	Insufficient protection in workplaces leading to potentially higher chance of infection among HCWs and therefore increased stigma	HCWs: (60.2%) working at university hospital, (25.6%) general hospitals, primary health care or centers (14.2%)	•Most common statements as causes of perceptions of fear of COVID-19 infection: fear of transmission of infection to families (98.5%), disease being highly transmissible (90.4%), no available vaccine (78.6%) or treatment (87%), fatality of the disease (82.1%), fear of entering COVID-19 isolation hospitals (86.5%), and stigma related to COVID-19 (66.3%) •Most common reasons stated by HCWs explaining higher susceptibility to COVID-19 infection than others were: PPE shortages (83.6%), crowded workplaces (61.4%) and ill ventilation (72%)	Cross-sectional survey
China (40)	Upper-middle income	Stigma around former COVID patients worsened mental health outcomes	Former COVID-19 patients including 13.3% medical staff (physicians and nurses who had been ill)	•Perceived discrimination associated with clinically significant PTSD symptoms, severe depression, and severe anxiety •Perceived discrimination was strong risk factor for all anxiety, depression, and PTSD and was always an important variable in predicting anxiety, depression, or PTSD	Cross-sectional survey
Ghana (41)	Lower-middle income	Lockdown measures including ban on social gatherings and lack of PPE for caregivers worsens mental health of cancer patents	Cancer patients	•Ban on social gatherings including religious activities important for support likely to worsen their mental health •Socializing and spirituality play a significant role in the health and well-being of cancer patients in Ghana	Case study
Italy (42)	High income	Social restrictions and limitations on mobility lead to worsening of symptoms for OCD patients	OCD patients	•Significant changes on severity of total OCD symptoms, obsessions, and compulsions from before the quarantine to quarantine period, suggesting overall worsening on all these outcomes •Those who could not work/study remotely during the quarantine, those living with a parent in the same house during the quarantine and those with contamination symptoms had a significantly stronger worsening on the severity of total OCD symptoms, obsessions, and compulsions from before quarantine to the quarantine period	Preliminary naturalistic study with semi-structured interviews
China (43)	Upper middle income	Insufficient knowledge among insomniacs leading to worsening symptoms caused by lockdown induced confinement	Insomnia patients (ages 18–65)	•Sleep latency, sleep duration, sleep efficiency and daytime function affected •During isolation period, patients have irregular work schedules and rest time. Patients sleep poorly at night and their social functions are affected during the day •During home isolation, patients worried and panicked which was mostly attributed to a lack of knowledge about disease prevention and control	Cross-sectional survey
China (44)	Upper-middle income	Due to strict lockdown measures, severe negative psychological impact on psychiatric patients with need to intervene and provide support and monitor and prevent increased suicide ideations	Psychiatric patients (ages 18 and above)	•Psychiatric patients scored significantly higher on the total IES-R, DASS-21 anxiety, depression, and stress subscales and, total ISI scores •More than one-quarter of psychiatric patients reported PTSD-like symptoms and moderate to severe insomnia. •Psychiatric patients were significantly more likely to report worries about their physical health, anger, impulsivity, and suicidal ideation •Improved access to telepsychiatry services, home delivery of psychotropic medications, online psychiatric first-aid resources, and infectious disease outbreak preparedness play a pivotal role in minimizing the severity of psychiatric symptoms experienced by psychiatric patients	Cross-sectional survey
India (45)	Lower-middle income	Inadequate knowledge among severely mentally ill and due to lockdown, decreased access to medication. Caregivers do not receive enough financial support and are overburdened	Severely mentally ill patients clinically stable before covid-19 and their caregivers (family): patients were from lower socioeconomic status (60.6%) with diagnoses being schizophrenia (59.1%), bipolar affective disorder (25%), major depressive disorder (12.1%), and schizoaffective disorder (3.8%). Ages were 18–55 years old	•Around 80%of patients missed appointments with mental health professionals in previous month and 22% stopped psychiatric medication due to the non-availability of medication and mental health professionals, lack of transportation, due to strict legal enforcement of lockdown •Two-third of patients lacked adequate knowledge of precautionary measures against COVID-19. Patients from lower socioeconomic status, low literacy levels, with inadequate social support showed less knowledge related to COVID-19 •Impairment was noted in sleep (37.9%), food intake (23%), and personal care (20%) and 29.5% showed re-emergence of previous psychiatric symptoms. •63.6% reported that they were experiencing verbal and physical aggression from others •30.3% caregivers reported increase in the burden of taking care of patients in addition to the burden related to other reasons, like the lockdown. •45.5% caregivers perceived inadequate social support during this period and 62.9% were facing financial difficulties during lockdown	Cross-sectional survey
Italy (46)	High income	Increased lockdown restrictions worsening mental state of severely mentally ill	Severely mentally ill and control subjects. the following diagnosis were included: schizophrenia spectrum; bipolar disorder; recurrent major depression (ages 18–70)	•Patients were four times more likely to perceive high COVID-19 pandemic-related stress and had 2–3 times higher risk of severe anxiety and depressive symptoms. •Patients with serious mental illness had lower economic status and higher rates of concomitant medical diseases. Actual perceived stress from COVID-19 outbreak and lockdown restrictions appears a strong predictor and mediator of the heightened risk of suffering from severe anxiety in patients with serious mental illness	Case–control
United States (47)	High income	Lockdown and home confinement increasing cases of gender-based violence with traditional support venues cut	Orthopedic trauma patients	•In 2019, 76 patients (26%) had a mental health diagnosis compared with 110 patients (43%) in 2020 •In 2019, 34 patients (11%) reported interpersonal violence vs. 51 patients (20%) in 2020 •Proportion of women presenting with fracture was higher in the 2020 group	Retrospective cohort study
Spain (48)	High income	Lockdown and home confinement lead to social isolation potentially worsening cognition and functioning due to limits on exercise and social interaction	Elderly (community dwelling with mild cognitive impairment or mild dementia)	•Living alone reported greater negative psychological effects and sleeping problems and some elderly changed living situation to have family as support network •Measures adopted to address negative experiences of lockdown included keeping informed, accessing health and social services, having support network that prevents risk of exposure to COVID-19 and guarantees food and medical supplies, daily routine with maintained sleeping habits and leisure activities, staying physically and mentally active with exercise, and preventing social isolation via technology	Cross-sectional
France (49)	High income	Lockdown limiting mobility and ban on social gatherings restricting ability to socially stimulate and physically workout—both critical to health	Elderly	•Despite decline in participation in group physical activities before quarantine, older adults expressed need to perform physical activity at home •Physical activity important for elderly to maintain level of independence, mental health, and well-being	Qualitative inquiry with semi-structured interviews
Spain (50)	High income	Lockdown limits community and family support for elderly and crucial for adequate resources for mental health available for elderly	Elderly (ages 60–80)	•Oldest of elderly and youngest of elderly showed no difference in psychological well-being and age only a negative impact on personal growth •Perceived-health, family functioning, resilience, gratitude, and acceptance had significant associations with both personal growth and purpose in life	Cross-sectional
China (51)	Upper-middle income	Lockdown measures including ban on social gatherings leads to reduction of outdoor activities and social interaction which are important for development	Children/students in grades 2 through 6	•Students who were slightly or not worried about being affected by COVID-19 had significantly lower CDI-S scores than those who were quite worried, with a decreased risk of depressive symptoms •Those who were not optimistic, compared with those who were quite optimistic, had significantly higher CDI-S scores, with an increased risk of depressive symptoms	Cross-sectional
China (52)	Upper-middle income	Lockdown interrupted education and increased pressure and stress especially on students preparing for entrance exams	Children/adolescents	•With increasing grade, proportion of students with depressive and anxiety symptoms increased •Scores for COVID-19 knowledge, prevention and control measures, and projections of COVID-19 trend higher among students without depressive and anxiety symptoms •Female students suffered greater psychological impact, and higher levels of stress, anxiety, and depressive symptoms	Cross-sectional

### Mobility Rights, Quarantine, and Lockdown

According to the international human rights law, restrictions of mobility including lockdown or mandatory quarantine due to public health emergency must be carried out for a legitimate purpose, based on scientific evidence, of limited duration, and respectful of human dignity (53). Quarantines are successful at limiting the spread of infectious diseases, but they introduce the side effects of increasing people's risk for psychological impact including suicide and other behavioral symptoms.

In our review, three studies reported rise in suicide incidence in several countries due to mobility restriction and lockdown. In Colombia, a national-level lockdown increased the risk of suicide in vulnerable populations and among those with pre-existing predisposing factors such as emotional problems, financial troubles, and job loss (29). One out of 13 adult Colombians reported high suicide risk; people experiencing depressive episodes or poor sleep quality including insomnia, had a higher risk for suicidal behaviors than the general population. In Pakistan, most of the investigated suicide cases were carried out by individuals who were socially-economically impacted by economic turmoil and experiencing financial troubles caused by the lockdown (30). Similar findings were reported in India where suicide cases increased as COVID cases increased, especially among individuals with pre-existing mental illness and those in poor socio-economic conditions (31).

Mobility restrictions were also found to be correlated with poor mental and social well-being in developed countries. In Italy, lockdown restrictions mediated other mental and behavioral symptoms such as anxiety in patients with serious mental illness (46). In the United Kingdom, since the start of their national lockdown, 9% surveyed participants reported experiencing psychological or physical abuse during the lockdown (32). About, 18% reported experiencing thoughts of suicide or self-harm in the first month of lockdown and 5% reported harming themselves at least once since the start of the lockdown. Reported frequencies of abuse, self-harm, thoughts of suicide, and self-injurious behavior were higher among women, black, Asian and minority ethnic groups, people experiencing socioeconomic disadvantage, unemployment, disability, chronic physical illnesses, mental disorders and COVID-19 diagnosis. Furthermore, a study in the United States found an increased number of orthopedic trauma patients reporting a mental health diagnosis post-quarantine compared to pre-quarantine as well as an increased number of patients reporting interpersonal violence as the reason for their injury (47).

### Shortage of Supplies and Equipment for HCWs

As part of the right to health, the International Covenant on Economic, Social and Cultural Rights obliged the governmental and health agencies to plan and optimize the use of personal protective equipment (PPE) among HCWs during public health emergencies such as COVID-19 (54, 55). This also includes providing them with appropriate training, education, and informational material that can prevent physical and mental harm from health care-associated infections.

According to the UNICEF and WHO, it is estimated that the majority of HCWs provide services and work in environments that lack basic infrastructure to support water, sanitation, hygiene and basic health care-related waste management (56). Lack of PPE has found to have negative consequences on HCWs' mental health. In a study conducted in Egypt, HCWs reported that crowded and ill-equipped workplaces and widespread shortages of PPE during COVID-19 pandemic increased their fear of getting a serious infection (39). These situations add emotional and mental burden for HCWs in attempting to self-isolate themselves from their families and communities (36). A cross-sectional study in Lebanon reported that quarantined HCWs suffered adverse mental effects such as anxiety, stress, and depression that increase under self-isolation because suspected COVID-19 infection (37). A similar study in Jordan found depression and anxiety to be more prevalent among HCWs than the general population. Specifically, pulmonologists and ENT specialists, who are at the frontline of the pandemic, scored higher in depression and anxiety surveys compared to other specialists (36).

Studies reported that fear in communities about the exposure to infection through interaction with HCWs expose them to fear-driven shunning and outright discrimination and persecution (38, 57). In Singapore, a study conducted among different HCWs found a higher perceived stigma level that was associated with higher levels of stress and post-traumatic stress symptoms. The study highlighted how internalization of stigma can reinforce avoidant behavior and social isolation which would increase traumatic stress symptoms, triggered by negative reactions from the surrounding community (38). Another study from Egypt found two-thirds of surveyed HCWs feared stigmatization and discrimination related to COVID-19 (39). The authors suggested that in collective societies and faith-based communities in Egypt, social stigma associated with COVID-19 can be addressed through education, clear announcing of health care policies, and launching stigma reduction programs. A study from China on former COVID-19 patients, including 13.3% medical staff, found perceived discrimination was associated with clinically significant PTSD symptoms, severe depression, and severe anxiety (40).

### Child Rights

According to the Declaration of the Rights of the Child (58), children's rights include protection, education, health care, shelter, and good nutrition. Numerous studies have found impacts of the pandemic on children's behavioral health, development and growth, physical health, and educational outcomes, with possible differential impacts by age and gender. In China, the national government imposed a reduction of outdoor activities and social interaction among the population, including children, out of fear of spreading the virus. This resulted in adverse outcomes in children's mental, social, and behavioral health. Specifically, it found that during home confinement, Chinese children, ages 7–11, who felt insecure and anxious, had a significantly higher risk of depressive and anxiety symptoms (51). Also, the closure of schools and educational institutions in China during lockdown disrupted the learning and educational process. It also deprived students of the sense of stability and normalcy that schooling provides. In a survey of 8,140 students in different educational stages, the proportion of students who reported depressive and anxiety symptoms was high, especially among those preparing for entrance exams which had been disrupted (52). The same study reported that female and male students differ in their perceptions of the psychological impact due to losing access to schools during COVID-19 with girls suffering from greater psychological impact, including stress, anxiety, and depressive symptoms (52). Also, specific facets of children's rights, such as health care, shelter, and nutrition, were also affected. Specifically, families that deal with lockdown and COVID-19 related financial stressors, struggle to provide basic needs and daily supplies, resulting in adverse mental health outcomes for family members such as stress, anxiety, and depression (59).

### Rights of Elderly Individuals

In recent years, there have been significant advocacy efforts supporting human rights of older persons including full respect for their needs, privacy, and health care (60). During COVID-19, elderly individuals were found to have less access to free movement including in open and public spaces which restricted their ability to exercise and engage in leisurely or other essential activities—deteriorating their mental health and well-being (49). In our review, we identified two studies in developed countries, and no studies related to elderly populations in developing countries. A study in Spain of community-dwelling older adults with mild cognitive impairment or dementia found that enforced lockdowns, curfews, and social isolation had more significant adverse psychological effects and exacerbated sleeping problems for elderly living alone (48). In contrast, another study found that the COVID stress-related factors, except for the loss of a loved one, were not statistically linked with the deterioration of psychological health among the elderly during stay-at-home orders; the participants showed resilience in managing COVID-related stress challenges due to sufficient personal resources (50).

### Disproportionate Impacts on Minorities and Psychiatric Patients

In accordance with the UN Pact on civil and political rights, national, ethnic, religious, or linguistic minorities (61) are meant to freely receive health services without any discrimination (62). In the United States, which has the highest number of confirmed COVID-19 cases as of August 2020 (Johns Hopkins University, 2020) (63), the populations that have been impacted the most disproportionately are racial and ethnic minorities. Prevalence for COVID-19 infection was reported higher in ethnic minorities than whites given inequality in health, welfare service access, and other existing social structural issues such as structural racism and discrimination, and this inequality and right violations may have led to higher mental health problems in racial and ethnic minorities. A cross-sectional study investigating differences between awareness about COVID-19 by race and ethnicity found African-Americans and Hispanics were less likely to be informed about the pandemic and effective prevention methods (33). These findings align with another study in India where patients with mental illness from disadvantaged backgrounds had less access to information via the internet, media, online health information (45). Unequal access to information along with lack of proper health care during lockdown jeopardize their physical and mental health.

Spreading of infectious diseases often fuels racism and xenophobic tendencies, especially against racial and ethnic minorities (64, 65). This results in a detrimental impact on the health well-being of disadvantaged minority populations and indirectly denies them access to medical care (66). A cross-sectional study linked xenophobia with mental well-being among ethnic/religious minorities: a survey of the non-Muslim Indian population found that fear, generalized xenophobia, and specific xenophobia, and collectivism negatively impacted their feelings and functional aspects of mental well-being (34). In another study, researchers screened the physical and mental health of minority migrants' workers in 140 areas across India, which included construction sites, relief camps, government hostels, and shelter homes during the COVID-19 pandemic. Their findings underscored that the economic, social, and environmental disadvantages were bothersome to the migrants and were worsening their mental health, undermining their resilience, and upsetting their quality of daily living (35). The impact was prominent among pregnant women as the interviewees who were feeling overwhelmed and concerned about their lack of access to health services because of the pandemic.

Similarly, unintended consequences of the strict legal enforcement of lockdown during the COVID-19, including lack of access to medications and mental health professionals and coupled with lack of transportation, negatively impacted treatment compliance among psychiatric patients. Specifically, in India, a study of severely mentally ill patients during lockdown found 80% of patients missed their appointments and failed to contact their mental health professionals, 30% showed features of relapse of symptoms during the lockdown, and 22% stopped their psychiatric medication with patients from lower socioeconomic status, low literacy levels, and with inadequate social support showing less knowledge related to COVID-19 (45). A similar pattern was observed among patients with Obsessive-compulsive disorder (OCD) in Italy. In a group of patients with OCD who had completed an evidence-based therapeutic path for OCD before the quarantine, there was significant worsening of OCD symptoms compared to the pre-quarantine period. The author hypothesized that the lockdown and limited access to mental health centers may have discouraged patients in from seeking help and delayed the needed interventions (42).

Psychiatric patients in China were found to show higher rates of anxiety, depression, and stress symptoms compared to the general population (44). Another study from China found insomnia patients had worse sleep latency, sleep duration, and sleep efficiency due to home isolation and insufficient knowledge (43). Due to limitation on mobility, cancer patients in Ghana were unable to engage in activities involving social gatherings, worsening their mental health (41). Even though psychiatric patients often experience high levels of social discrimination (67), in this review, a study in China reported that patients with mental illnesses had not been experiencing additional discrimination during the COVID-19 epidemic (44). Though, in India, self-isolation and stay-at-home orders forced many psychiatric patients to live in unsafe homes and increase their exposure to domestic violence. In India, ~63.6% of psychiatric patients reported that they were experiencing verbal and physical aggression from others and 30.3% of their caregivers expressed a feeling of the excessive burden of taking care of patients in addition to the burden related to other reasons, like financial issues related to the lockdown (45).

## Discussion

### Overview

The review identified human rights violations that are associated with adverse mental health outcomes such as mobility restrictions including quarantines and lockdowns, shortages of supplies and equipment, stigma, xenophobia, and discrimination, losing access to schools and proper education, lack of access to information, and inequalities around access to quality mental health services. In addition, the findings of this review underscore several points in advancing our understanding and knowledge of human rights restrictions and violations and their association with mental health well-being during the COVID pandemic. *Given these findings, we would like to make a case that human rights of individuals and vulnerable populations must be protected during this pandemic and given that mental health is one of the fundamental rights, it needs prioritization at the public policy level* (8). *Human rights and mental health are such delicate entities that at times public health and public policy actions might compromise larger interests of the most afflicted or vulnerable populations*. Evidence-based policies may not always be representative of all members of the community. Therefore, ethical and rights-based approaches to ensure no added harm or disenfranchisement of individuals/groups is a sentiment that needs to be acted upon post-pandemic.

### Summary Findings Around Vulnerable Groups

From the articles reviewed, 24 identified studies were published between April and July 2020. This is consistent with the fact that the health emergency measures in response to COVID-19 were declared worldwide in March 2020 and therefore most relevant studies for this review would take place in the following months. Many of the identified studies were conducted in developed countries and there may be variation in the understanding of human rights and legislations across countries. See Table 2 that enlists Key UN human rights protocols and policy documents for diverse populations. The definition, understanding and agreement on vulnerable populations might also vary from one country context to the other. The understanding, development and functioning of public mental health systems might also vary in different geopolitical contexts. Previous studies found that many developing countries lack the basic legal framework to protect patients suffering with mental illness as well as a recognition that there is an absence of specific human rights policy during health emergencies to guide mitigation and protection measures (13). Both observations lend evidence to disproportionate impact on vulnerable key populations. The intersectional nature of multiple minoritized identities coalescing together such as being a migrant, disabled, and an adolescent girl—their complexity, need for comprehensive policy action—cannot be under-emphasized here.

**Table 2:** Key UN human rights protocols and policy documents for diverse populations.

**Legal/ethical policies**	**Guidance and recommendations**
Emergency measures (17)	State of emergencies should: •Be temporary in scope, not used to stifle dissent, transparent, and least intrusive to achieve goals •Have safeguards such as sunset or review clauses and allow independent review and legislative scrutiny •Not violate non-derogable rights and use deprivation of liberties as penalty only as last resort and be reasonable, lawful, and appropriate •Not arbitrary, unreasonable, necessary, proportionate to interest at stake, non-discriminatory, without arbitrary enforcement, and justified •Support core economic and social rights of people •Make sure law enforcement is compliant with international standards and human rights violation by them are met with swift investigation and justice
Human rights of migrants (18, 19)	To respect the human rights of migrants, states should: •Put in place legislative, policy, administrative measures to ensure timely and effective access to health facilities regardless of immigration status •Have provision of essential services separate from immigration enforcement and have social protection measures against unemployment available •Counter stigma related to racism and xenophobia through measures to prevent and address •Make available information on prevention, diagnosis, and treatment of COVID in languages migrants can understand •Take specific actions need to be taken to protect health of migrants in inadequate and unsafe conditions •Include migrant children in policies for access to education and think of innovative ways for access to education •Guarantee tightened border controls is non-discriminatory, confidential, and handled with dignity—and not imply indefinite detention •Release migrants in detention and give services and non-custodial alternatives to protect both migrants and staff •Guarantee labor rights of migrant works, and especially for those working in essential sectors •Include migrants and their families in economic recovery policies •Guarantee right of all migrants and their families to return to the country of which they are nationals
Women's human rights (20)	States should: •Recognize women are 70% of the frontline HCWs and therefore need adequate PPE and protection from stigma, safe and confidential access to health services, and protection of sexual and reproductive rights •Place special emphasis and attention to make sure girls continue their education particularly in LMICs •Declare protection structures and services for victims of gender-based violence as essential •Update referral pathways to reflect changes in available care facilities, ensure sufficient safe shelters •Adequately resource hotlines and other support and reporting mechanisms •Raise awareness via channels victims may seek help through and exempt that fleeing violence from punishment
Action on mental health (21)	States should •Consider mental health as essential components of national response and craft communication to be aware of potential impact on mental health •Ensure the widespread availability of emergency mental health and psychosocial support such as scaling up investments to remote services •Declare in-person care for severe mental health disorders as essential •Ensure social connectedness for older adults and other vulnerable populations in confinement through support of community action strengthening cohesion, solidarity, and healthy coping to reduce loneliness •Protect and promote the human rights of people with severe mental health conditions and psychosocial disabilities, for example, by monitoring whether they have equal access to care for COVID-19 •Develop and fund the implementation of national services re-organization strategies that shift care away from institution to community services •Ensure mental health is part of universal health coverage
Human rights of persons with disabilities (22)	States and stakeholders need to: •Prohibit denial of treatment on basis of disability, ensure priority testing, and promote research on impact of COVID on disabled •Identify and remove barriers to treatment and ensure the continued supply and access to medicines •Closely consult with and actively involve disabled people and representative organizations •Conduct training and awareness-raising of health workers •Refrain from blanket prohibitions of leaving the home and instead create exemptions for persons with disabilities to be outside
COVID-19 and civic space (23)	States should: •Create avenues for participation and feedback and reach out to most at-risk (e.g., women, older persons, disabled) •Maintain existing channels of civil society participation •Uphold freedom of assembly and ensure the limiting of the exercise of that right is only and strictly tied to protect public health •Respect right to privacy and adequate safeguards and accountability ensured with any surveillance proportional, lawful, and necessary •Respect freedom of expression must be respected and not engage in limitation on access to relevant data, censorship, or criminalizing journalistic activity •Not penalize expression based on vague concepts which are not compatible with requirements of legality and proportionality
Human rights of older persons (24)	To ensure the well-being of the elderly, states should: •Prioritize testing of vulnerable populations including older adults living in long-term facilities •Ensure continuity of adequate care services such as mental health services, palliative and geriatric care, including through support for caregivers •Strengthen services to prevent and protect older persons, particularly older women, from violence and abuse, such as domestic violence and neglect •Ensure visitor policies in residential care facilities, hospitals and hospices balance protection of others with need for family and connection •Assess needs, particularly those more isolated or with cognitive decline/dementia to provide support, including mental health and psychosocial
	•Do not stigmatize them and avoid stereotyping. Avoid labeling older adults as uniformly frail and vulnerable or words with negative connotations •Adopt socioeconomic relief measures and social safety nets, such as guaranteed access to food, water, essential goods and services and basic health care
Human rights of children (25)	To minimize the impact of COVID-19 on children, states should: •Rebalance combination of interventions to minimize impact of standard physical distancing and lockdown on children in low-income countries and communities and expand social protection programs to reach the most vulnerable •Prioritize the continuity of child-centered services, with focus on equity of access—particularly in relation to schooling, nutrition programs, immunization and other maternal and newborn care, and community-based child protection programs. •Provide practical support to parents and caregivers, including how to talk about the pandemic with children, how to manage their own mental health and the mental health of their children, and tools to help support their children's learning
Human rights of minorities (26, 27)	States should: •Ensure equal access to health care and eliminate any discriminatory practices against racial or ethnic groups •Prioritize access to free or affordable testing, medications and needed procedures •Involve communities and representative associations in designing and implementing health programs •Ensure racial or ethnic minorities are not disproportionately controlled, harassed and profiled by law enforcement authorities •Ensure the right of individuals to an effective remedy against the perpetrators of acts of racial discrimination, including when such acts are committed by law enforcement officers or other State officials •Ensure online learning does not exacerbate existing racial inequalities, and bridge digital divide •Mitigate disproportionate impacts of violation of the right to adequate housing through measures that include providing direct financial assistance, and enacting a moratorium on evictions due to arrears •Ensure food, water, and sanitation facilities are accessible and available in quantity and quality sufficient to satisfy needs of all •Adopt fiscal stimulus and social protection packages aimed to mitigate longer term economic and social consequences of pandemic
Human rights of persons in detention (28)	States should: •Address prison overcrowding, prioritize release of individuals of those who are children, persons with health conditions, low risk profiles, and persons with imminent release dates •Ensure that rationing of health responses and allocation decisions are guided by human rights standards based on clinical status and do not discriminate based on any other selection criteria, such as age, gender, social or ethnic affiliation, and disability to minimize creating avoidable rise in anxiety and stress levels, especially among children and the elderly

*Key human rights protocols and policy documents relevant to this review are summarized in this table*.

In our review, a strong association between the adverse mental health outcomes and human rights restrictions such as quarantine has been well-documented, a clear finding in our review with anxiety and mood disorders being the most common conditions impacting populations (68). Additionally, *countries with higher-than-average rates of stress-related symptoms pre-COVID may continue to experience increases if effective measures are not implemented which necessitates a collective plan of action*.

Findings of adverse mental health outcomes following human rights violations were reported among vulnerable subgroups such as children, girls, elderly, and racial/ethnic minorities. The two studies that examined the mental health-related adverse outcomes associated with human rights violations found significant deterioration in children's mental, social, and behavioral health due to restriction in daily activities and movement (51, 52). It is important to note that the lockdown and social distancing could cause children—and especially children with mental disorders including developmental disabilities or physical or psychological comorbidities—to be prevented from fulfilling their psychological needs and developmental milestones leading to underdevelopment (69). Furthermore, home confinement can increase child abuse as pre-pandemic the vast majority of abuse was reported by staff working at institutions which were temporarily closed raising concerns that child abuse will go unnoticed, leading to higher child morbidity and mortality and long-term negative developmental consequences (70). The United Nations Population Fund (UNFPA) estimates, due to the COVID-19 pandemic, 42–66 million more children potentially falling into poverty, and 13 million child marriages by 2030 that could otherwise have been averted, and 2 million more cases of female genital mutation by 2030 that could have been avoided (71, 72). Child marriages, child labor and such practices can flourish unchecked during this protracted pandemic. This is also an example of practices and cultural traditions that would yield in harmful short and long term mental health outcomes for the vulnerable children.

In this review, we found that girls suffered varied mental health outcomes due to violations of their human rights via losing access to school (52). These are consistent with what is reported by WHO (73), as existing gender inequalities are exacerbated by COVID-19 which affect both genders differently (74). The realization of girls' human rights and their well-being, especially during emergencies, have not always been accorded priority attention (75). Furthermore, females' access to sexual and reproductive health services is likely to be affected (76). In our review, difficulties were reported by pregnant women in accessing basic prenatal services during the pandemic due to closure and movement restrictions (35) and a previous report found that stay-at-home orders may contribute to women's vulnerability and considered a risk factor for gender based violence (74). Mobility restrictions can increase domestic violence and in our review a study from the United States found an increase in orthopedic trauma patients reporting interpersonal violence post-lockdown compared with pre-lockdown with an increase in fractures in female patients indicating an increase in gender based violence (47). Additionally, a UN human rights report alluded to rise in gender based and domestic violence due to economic and psychosocial challenges (76). *We do strongly recommend national level action on mitigating the harms caused by violence on women and girls as domestic violence is both a cause and consequence of mental ill-health* (77). A gender responsive training for health care workers, police, law enforcement officers and social protection workers would be critical in reducing the disproportionate burden of mental ill-health that will fall on young girls and women exposed to abusive environments.

Our review found HCWs suffered negative consequences as a result of shortage of supplies and equipment as well as stigma (39). Other reports have also found mistreatment because of stigma toward HCWs such as the denial of public transport, eviction from homes, and assaults when carrying out duties negatively impacted their mental health (75). To mitigate stigma, proper health education is needed to target the public to prevent social harassments of HCWs and COVID-19 survivors.

Regarding elderly's rights, two studies examined the association of human rights violations and mental health outcomes among elderly people in developed countries (48, 50). Common characteristics across the studies that were indicative of human rights violations were: enforced lockdown, curfews, and restricted the ability to exercise and engage in essential activities. No identified study examined old age discrimination or factors that directly contributed to autonomy of elderly individuals. However, other reports have noted that well-intentioned but ageist public messages can cause the elderly to be marginalized and to mitigate this, countries can use inclusive language and avoid negative emphasis on risk (78). Furthermore, to lessen potential adverse mental health impacts on the elderly—especially those with mental disorders or predisposed to feelings of loneliness—due to home confinement and curtailing of mobility rights, countries can engage in promoting social connection in public health messaging, mobilizing resources from family members, and giving help via community-based networks (79, 80). One of the moral imperatives we have as a global community is to strengthen mental health via reduction of increasing social isolation in the face of this protracted pandemic.

Studies have noted with concern adverse mental health outcomes due to human rights violations including lack of access to accurate and up-to-date information among racial and ethical minorities (33). Our review also found xenophobic viewpoints were correlated with negative well-being for both holding those viewpoints and those targeted which have co-incited with underreporting and biased media coverage as well as misinformation targeting religious minorities (34). In our review, a study has revealed an additional model of ecology of health and sickness based on social determinants of health among migrant workers which are one of most marginalized and stigmatized population in LMICs (35). It has also been noted that refugees and immigrants in detention centers face an increased likelihood of developing post-traumatic stress disorder as many refugees associate economic hardship, food and medicine shortages with a threat to life alongside viewing military presence to enforce restrictions as a threat and not a protection (81). There has been little-to-no scholarly work on this subject though police brutality has been on the rise in context of enforcing stay-at-home and mask-guidelines (82). *Concerns have been raised over the disproportionate impact of over policing in enforcing lockdown measures toward minority communities* (83). *There have also been reports of physical beatings or killings over breakings of curfew laws* (84) *which is a violation of human rights* (85). The mental stress and trauma associated with such events would become subject of further inquiry as time moves on but sensitivity training and ethical psychosocial response needs to be embedded within judicial and armed services assisting emergency response. Racial, ethnic and religious discrimination remains an area with very limited evidence around how mental health field can be leveraged to promote equity and social justice focused messages but also ways of providing care to aggrieved populations.

Human rights restrictions and violations have significant impacts on treatment outcomes, but higher impacts on patients with mental disorders given pre-existing mental health condition and social ostracization due to stigma. Research suggests that patients with mental illness are suffering from worsening of their pre-existing condition and difficulty in maintaining medication adherence because of restriction of mobility and lack of access to medications (42, 45). This was coupled with increased experiences of verbal and physical violence at hands caregivers. It is important to limit the abuses of human rights of those with mental health conditions especially by curtailing marginalization and stigmatization and increasing resources toward providing services (86). Article 11 of the Convention on the rights of individuals with disabilities (CRPD) stipulating that all necessary measures to ensure protection and safety of persons with disabilities in situations of risk should be pursued (87). Furthermore, it is vital to ensure the well-being of caregivers of vulnerable individuals. This is especially pertinent in a disability setting as the caregiver's well-being is also important for the well-being of their charge or family member. A side effect of the lockdown in Europe on people with disabilities has been a massive harmful impact with reductions, early discharge, and the stoppings of admissions to rehabilitation expected to lead to an increased rehabilitation demand after the emergency subsides (88). *Despite the boom in digital health during the COVID-19, no reported studies examined the role of engagement of technology with available services in mitigating the structural barriers to getting mental health care during the COVID pandemic*. For example, concerns have been expressed that digital health services may not assist in decreasing inequity due to factors such as limited access of digital health services for those in poverty (89). This is an area in need of serious research and policy level attention. Resource allocation in providing quality digital and telehealth services, online learning or improving access to services for high risk groups also needs urgent prioritization.

### Upholding Global Rights Conventions and Further Recommendations

UN committees that monitor nation states human rights actions include UN International Covenant on Civil and Political rights (ICCPR) (90), Committee on Economic, Social and Cultural rights (CESCR) (55), Committee on Elimination of Racial Discrimination (CERD) (91), Committee on All forms of discrimination against women (CEDAW) (92), Committee against Torture (CAT) (93), Convention on the rights of the child (CRC) (94), and CRPD (95). In the context of emergencies, these committees advocate for right to health, human rights in health systems as well as upholding of human rights through health (96). There are a number of guidelines in place as seen from Table 1 on key recommendations mostly from the Office of the High Commissioner around COVID-19 and vulnerable populations. The UN recommendations and guidance around COVID stipulates a public health approach to managing the pandemic impact based on equal rights, opportunity, and valuing freedoms especially of the most-at risk populations. Many share similar recommendations such as advising for more investment into these vulnerable populations to ensure the negative impacts of the lockdown—including quarantine, social distancing, economic fallout—are lessened and disproportionate limitations around access to services and rights due to structural inequities are addressed. It is important to consider these as they provide valuable insight in how to minimize the consequences of the COVID-19 pandemic response and its response which embeds human rights informed care and risk mitigation pathway. However, there needs to be more emphasis on strategies that would address lockdown, public health action associated impacts and their mental health outcomes, and the need to prioritize mental health and well-being holistically. Given that the pandemic is going to take us through several waves of infection spread and containment, it is critical for this rights based approach to embrace short and long term mental health consequences and mitigation strategies.

If we are to achieve a shared vision of protecting the population from adverse mental effects due to the pandemic and necessary pandemic responses, policy makers would benefit from an increased focus on strengthening leadership and governance, finance mechanisms, and developing programs and policies that include vulnerable populations (97). *For LMICs, it is critical for the service provision be focused on accessible and equitable evidence-based community care models corresponding with the existing mental health capacity to deliver care, train existing primary care staff to cater to increased mental health needs, implement prevention and promotion programs tailored to local needs, and support civil societies and employers to address the increased burden of mental illness*.

Furthermore, mental health can be supported in the form of investment in the development of virtual platforms and telehealth to provide psychoeducation, self-guided low intensity interventions, alongside peer, and specialist-supported e-therapies. These can be embedded within integrated electronic health records and service platforms (98). Task shifting to provide psychosocial and peer-based support will also be useful such as using lay volunteers trained to provide human connectedness to address physical distancing and resultant isolation- a strategy well-adopted in LMICs. These approaches will also be beneficial in HICs in that effective management of the mental health consequences of the pandemic in historically marginalized communities requires a multilevel, community-wide approach led by culturally sensitive mental health professionals (99).

### Improving Care for All

Regarding vulnerable and at-risk populations, *there is a specific need to pay attention to their mental health as many of the inequities, access and service issues have longstanding history prior to COVID-19, and the pandemic has only further exacerbated them*. Human rights violations in the mental health context remain significant throughout the world and cannot be explained by a lack of resources alone (100). WHO QualityRights initiative aims to reform human rights within mental health field and has developed policies and sensitization strategies to carry out system level strengthening of a holistic response to mental illness keeping our right to dignity, freedom and health at center (101). This thinking has been part of the WHO mental health treatment gap action program (mhGAP) framework to champion human rights informed thinking and rally for mental health as a core human right. Various fundamental human rights are interdependent therefore undermining one leads to a poor impact on others (102). This means that all human rights should be addressed together, including mental health.

To help strengthen mental health systems researchers should focus on developing robust information systems able to be enhanced by linking with other data sources to run predictive models using robust informatics methodology. A focus can also be placed on community-based interventions to address the COVID-19 mental health issues in integrated approach alongside innovative digital solutions (97). There is an urgent need for research to address how mental health consequences for vulnerable groups can be mitigated under pandemic conditions, and on the impact of repeated media consumption and health messaging around COVID-19 (103).

*Addressing human rights can be a helpful way to mitigate the impact of mental illness on populations especially during public health emergency. Policy and frameworks to address different aspects of human rights can complement the various form of health services to enhance the mental health well-being during pandemics*. However, effective implementation of the medico-legal and ethical aspect of the human rights will rely on the extent to which the research proved a direct association between violations and adverse mental health outcomes and how the implementation will be applied through public policy. Going forward this understanding may help to minimize the mental health risk associated with violations of human rights during health emergencies and provide the policy makers useful directions on how to address discrimination and human right violations in guidance, policies, and practices (104). This review we hope has provided findings that set the foundation on how to integrate human rights as an integral part of public health response keeping mental health as a core thematic. In addition, the review provides pointers to ethical guidance when dealing with vulnerable populations especially during public heath crises (see Table 3 for further recommendations and overview of prominent findings). To target concerns about human rights violations during pandemics, policy makers should work with mental healthcare providers and target populations to ensure that suitable emergency health measures are provided through human rights lenses. This collaborative approach can pave the road to continue engaging or increasing engagement of different stakeholders in different sectors during stressful periods.

**Table 3:** Emerging findings and recommendations going forward.

**I OVERALL KEY FINDINGS**
• Fundamental human rights of the elderly, individuals living with mental illness or disabilities, and other vulnerable populations are disproportionately affected due to COVID restrictions. These rights violations are compounded by significant levels of discrimination and stigma
• HCWs experienced stigma and discrimination in addition to neglect in their working conditions putting them and their families at risk
• Most of the empirical studies covered COVID-19 restrictions which, when protracted, lead to significant human rights violations
• Gender based violence and income inequities increased as a result of lockdown contributing to loss of livelihood and socioeconomic strains and disproportionately affecting women and girls
• Limited access to enhanced alternative services (such as m-learning or e-health support) whether educational, vocational or health to populations most disproportionately impacted by the pandemic
• Significant loss of education and social protection to children in vulnerable contexts due to closure of schools and other related servicers
• Socioeconomic and health costs of worsening mental health of vulnerable individuals—the motto of “do no harm” flouted during pandemic restrictions meaning restrictions meant to protect should not worsen health or harm
**II OVERVIEW OF IMPACT AND RECOMMENDATIONS TO POLICY MAKERS AND RELEVANT STAKEHOLDERS**
•*Mental health is a fundamental human right* and irrespective of race, class, gender, ethnicity, or age, needs to be prioritized during emergencies. If considerable populations become scarred as a result of this pandemic, their worsening mental health would deteriorate their own and nations' recovery from the pandemic
•*Health systems strengthening approach:* human rights violations tend to increase when systems are under-resourced and poorly managed. A holistic health system strengthening approach and adequate response to human rights is needed
•*Equity and justice through health:* one of the ways of upholding CRPD and CRC and related conventions is to address systematic, structural inequities by providing health services and prioritizing multidimensional needs of the vulnerable
•*Protect civil liberties*. It is also important to inform the affected population of the exact substantive, territorial and temporal scope of the application of the state of emergency and its related measures as suggested by the ICCPR guidelines. Additionally, it is important to value individual and community's right to expression of their concerns and voices
•*Legality of enforcement of bans/closures/restrictions:* As recommended by ICCPR and other international rights guidance, legality of state action and role of state actors on lockdown, restrictions and delays in services/opportunities needs to be kept in mind. Protracted restrictions run the risk of increasing levels of apathy and increasing violations by people in charge
•*Human rights violations would lead to increased mental distress:* abrasively implemented state actions can make individuals and families prone to mental distress and illness; these effects can be long lasting
•*Have a pandemic and post-pandemic human rights approach infused with mental health:* building back nations and economies requires developing community level solutions through champions who can address needs and identify strategies to build back better. State level funding and resource allocation is needed to address mental health during and after the pandemic
*Recommendations include –*
•*prioritizing protecting lives and valuing marginalized and vulnerable populations' interests, helping people anticipate and address their health, and improved service access and livelihood challenges* •*address discrimination and stigma in time, rapidly train government and civil services in what needs to be a human right informed response to the pandemic* •*keep an eye on efforts to reducing inequality and disparities during and post-pandemic to make systems stronger*
*For certain specific vulnerable populations:*
°*Improve access to telepsychiatry services, home delivery of medications, and online psychiatric resources to minimize relapse of or worsening of symptoms by psychiatric patients* °*Offer moral and financial support to HCWs, especially those under quarantine* °*For elderly, adopt measures to ensure they are keeping informed, accessing health and social services, and guarantee food and medical supplies* °*Use trusted sources in racial or ethnic minority communities to disseminate information and ensure job security* •*Create a multi-stakeholder dialogue and discussion for policy action so it is well-informed, consultative and transparent in decision making, based in equity, with access and needs of most disenfranchised prioritized, with human rights elements relevant to COVID made part of mission statements of organizations, and learns from success stories within your own country and from other countries* •*Actions to embrace for HICs:* racial, ethnic and religious minorities are more vulnerable and so upholding their civil liberties and quality services and enhanced access is critical to reduce disproportionate ill-health burden. There needs to be a coordinated response between resourceful governments on supporting other countries by example and leading mobilization of action around inequity, stigma and discrimination both of mental illness as well as COVID-19 related fear and phobic responses. Gender and income inequity remain the key areas of action •*Actions to embrace for LMICs:* enhanced investment and improved access to quality health services including mental health. Universal health coverage (UHC) needs to be prioritized and mental health care has to be an integral part of the UHC. Countries should actively develop policy and programmatic response that would reduce gender and income inequality. Given that the pandemic would impact a large number of informal workers in LMICs, services need to be offered without discrimination. Stigma and discrimination have to be actively addressed and focus on key vulnerable populations should receive policy and programmatic prioritization
**III RECOMMENDATIONS TO MENTAL HEALTH PRACTITIONERS**
• Giving a voice to the prioritization of psychosocial needs of vulnerable and at-risk highly marginalized populations including a trauma-informed approach. Development of low intensity interventions that can be rolled out with strategies such as task-sharing and task-shifting. Psychotherapy support should be promoted through social works and community volunteers
• Integrate system level human rights approach in delivering essential health services and in specialist health care: training health personnel, teachers, social, and other essential workers in basic psychological first aid and human rights-based service provision and system thinking
• Recognize how inequities and inequalities have exacerbated global burden of mental disorders and actively develop policy, programmatic and research level instruments to combat these disparities and improve mental health conditions of populations. Identify conceptual models and theories that guide intervention development targeting improved mental health outcomes, equity, emancipation, and social justice as key themes
• Develop solutions such as public information programs to address and caution against stigma and discrimination and use media platforms to disseminate anti-stigma and discrimination messages

Although this review provides evidence that is valuable for addressing human rights perspectives while providing mental health services to different population, future research should (1) consider that some countries are still not signatories of the UN universal declaration of human rights and countries may have a different interpretation of what counts as a human rights violation; (2) countries also have different legal systems to define and enforce human rights measures; (3) there is need to arrive at a consensus on defining of human rights restrictions and violations in mental health and how it should be identified and quantified; and going forward through different UN human rights conventions as instruments to make that change; (4) adequate reporting of human rights restrictions and violations especially among vulnerable populations should be better etched out and this should include adverse mental health outcomes.

### Strengths and Limitations

This review followed the guidelines for a rapid review. The review highlights human rights violations influencing the mental well-being in impacted population. We summarize the agreements and global or national policies that enforce human rights during emergencies as a reminder of what we ought to be looking out for and values countries need to hold themselves accountable to. Our findings contribute to a broader understanding of the impact of COVID pandemic on population health. This review does have some limitations, however. The research team tried to identify and include as many published papers as possible; however, because of the novelty of the topic, the defined articles were limited and the rapidly evolving nature of the pandemic and associated scholarly literature could have caused some relevant articles to be missed. There is difficulty in creating a comprehensive list of human rights constructs as they are different based on factors such as target population and country of interest (105). Also, articles that were not data-driven were not included in our analysis, which may have impacted results of this review. Nevertheless, this approach was applied to ensure peer-reviewed articles were included which strengthens our results. Moreover, the study team attempted to group the results based on the themes that reported in the WHO report (84). We only included articles that addressed the research questions of focus of the review and therefore, generalizing the results on different setting and population should be done with caution.

## Conclusions

Finally, this review also acknowledges the implications for guideline and policies on different populations and therefore, (1) efforts to improve engagement of policy makers may be beneficial to address mental health outcomes during public health emergency and (2) civil society and human rights champions (including lived experience representatives), health care providers, and policy makers should work together to identify the policy, services, and interventions that enforce human rights which have a lasting impact on mental health of key populations during emergencies. We will be better served to understand the tradeoffs and the reciprocating relationships among physical health, mental health, human rights, and individual rights, that there could be better statements in global reports of values-driven, shared objectives in light of these tradeoffs and relationships, and a more thorough consideration of many relevant areas of science (including but not limited to, infectious disease) to establish a policy that strikes the most acceptable balance.

## Author Contributions

MR and MK developed the study protocol. RA helped edit the protocol. MR, RA, and MK extracted the data and compiled the initial list of studies. MR and RA along with MK wrote the manuscript. All authors read, edited, and approved the final version of the paper.

## Conflict of Interest

The authors declare that the research was conducted in the absence of any commercial or financial relationships that could be construed as a potential conflict of interest.
